# Prevalence estimates of major neurocognitive disorders in a rural Nigerian community

**DOI:** 10.1002/brb3.481

**Published:** 2016-05-05

**Authors:** Adesola Ogunniyi, Akindele O. Adebiyi, Ade B. Adediran, Olaide O. Olakehinde, Akeem A. Siwoku

**Affiliations:** ^1^ Department of MedicineCollege of MedicineUniversity of IbadanIbadanNigeria; ^2^ Department of Community MedicineCollege of MedicineUniversity of IbadanIbadanNigeria; ^3^ Department of PsychiatryCollege of MedicineUniversity of IbadanIbadanNigeria

**Keywords:** Cognitive disorders, epidemiology, geriatrics, low and middle income countries, neurosciences

## Abstract

**Objective:**

There is paucity of information on major neurocognitive disorders in sub‐Saharan Africa where the number of individuals with neurocognitive disorders is expected to increase due to demographic transition. This study aims to report on the prevalence estimates of dementia and MCI (mild cognitive impairment) in a rural community in southwest Nigeria.

**Materials and Methods:**

This was a two‐stage cross‐sectional study of persons aged 65 years and above resident in Lalupon community, Oyo State. The Identification and IDEA (Intervention for Dementia in Elderly Africans) Study Questionnaire was used for initial screening by trained community health care workers, utilized followed by cognitive assessment using the validated IDEA cognitive screen. Functional and cognitive assessment of selected individuals was carried out during the second stage. Information obtained was used for consensus diagnosis and participants were categorized into normal, MCI and dementia using standard criteria.

**Results:**

Six hundred and thirteen participants completed the study with 111 (18.1%) diagnosed as MCI while 17 (2.8%) had dementia. The age‐adjusted prevalence estimates were 18.4% (95% CI: 14.9–21.9%) and 2.9% (95% CI 1.6–4.4%) for MCI and dementia, respectively. Probable Alzheimer's disease and amnestic MCI predominated. Individuals with dementia were older than both MCI and normal cases while those with MCI had significantly fewer years of schooling than the other diagnostic categories.

**Conclusion:**

Almost one out of five older persons in Lalupon community had major neurocognitive impairment with MCI being six‐times more common than dementia. Alzheimer's disease was the most common dementia sub‐type.

## Introduction

Dementia and MCI are major neurocognitive disorders which pose considerable public health challenges worldwide (World Health Organization, 2012). Dementia is dehumanizing in its advanced stages. MCI occupies the intermediate stage in the continuum of cognition and is considered as the leading edge for preventive strategies (Unverzagt et al. 2007). The projected increase in the number of dementia cases in the coming decades in LMIC (low and middle income countries) according to a global Delphi consensus study (Ferri et al. 2005) is worrisome particularly in SSA (sub‐Saharan Africa) where many health care systems are under‐resourced, and communicable diseases like HIV and tuberculosis remain pervasive. The strain imposed by the disease on caregivers is quite considerable with loss of income, and psychosocial stress (World Health Organization, 2012).

There is paucity of information on neurocognitive disorders in SSA, and to date, there are less than twenty community‐based studies (Olayinka and Mbuyi 2014). In recent systematic reviews of published studies in SSA, the prevalence rates of dementia varied between <1% and 10.1% (Ogunniyi and Akinyemi 2010; Mavrodaris et al. 2013; Lekoubou et al. 2014; Olayinka and Mbuyi 2014). Very few studies have documented the prevalence of MCI (mild cognitive impairment) in communities (Guerchet et al. 2009; Mavrodaris et al. 2013; Olayinka and Mbuyi 2014). In a recent publication from Hai District, Tanzania, MCI prevalence of 7.0% was reported with about a third of the diagnosed individuals progressing to dementia over a 4‐year period (Paddick et al. 2014). Correlates of cognitive impairment, not dementia were documented in a study in Ibadan (Baiyewu et al. 2002). Therefore, more community‐based studies are necessary in SSA to provide baseline data on these two major neurocognitive disorders and for observing changing trends. Such studies could facilitate the search for environmental risk factors when communities with high and low rates are compared and for planning community‐based interventions. In this article, we report our findings on the prevalence estimates of dementia and MCI in a rural community in South‐west Nigeria.

## Materials and Methods

Ethical approval for the IDEA study was obtained from the University of Ibadan/University College Hospital, Ibadan Health Research Ethics Committee as well as from the Oyo State Ministry of Health, Ibadan. The study site was Lalupon, a community of 15,854 inhabitants (according to the 2003 National Census) and situated in Lagelu Local Government Area of Oyo State, Nigeria. The town is located about 20 miles northwest of Ibadan, the capital city of the State. It is inhabited by Yoruba whose main occupations are farming and trading. The area has primary health care facilities and is regularly visited by staff of the Department of Community Medicine of University College Hospital Ibadan. Nigeria is currently in the LMIC category. The Local Government Chairman and the Health Committee were briefed about the study and administrative approval was also obtained. Lastly, the representatives of the elders in Lalupon community were contacted and their consent obtained before the study commenced.

### Study design

This was a community‐based study that involved door‐to‐door visit and face‐to‐face administration of the screening questionnaire to eligible participants and separately to their informants. The three wards (Lalupon I, II and III) in which study was domiciled were designated as clusters and all houses were visited in these wards to identify eligible participants. Thus, a total sampling of the selected communities was done. Consent was obtained from each participant before the interaction and assent from a close relative for those who had dementia. Those that were educated provided signatures while for those with no education, thumb‐printing was done. The screening phase took place between May 1 and October 31, 2013. Our inclusion criteria were as follows: persons of either sex aged 65 years and over, consent and clear consciousness. We excluded individuals aged <65 years, those that refused to give consent, older persons with clouding of consciousness, severe speech disturbance and those on treatment for severe psychiatric illness. All the recruited participants were of Yoruba ethnicity and there were no refusals. Since records of births were not readily available, we used the validated method of historical landmarks to determine the ages of participants (Ogunniyi and Osuntokun 1993).

Structured interviews of the subjects and reliable informants were carried out in each participant's home. For the latter, preference was for the spouse or for someone who stayed in the same house with the study participant. However, some participants were assessed in the Community Health Centre either because they were not present at home at the time of their first visit or on request for convenience. Four Community Health Care workers were trained and certified on the administration of the screening questionnaire. Their Interrater reliability was 100% using kappa statistics for all items after repeated training and practice of the interviewers.

### First stage assessment

The IDEA study screening questionnaire was designed to obtain information on impairment, mode of onset and pattern of progression of impairment in the following cognitive domains: memory, language, judgment, reasoning and personality. We obtained information on any impairment in financial or social activities, home care, looking after grandchildren and personal care. The informants' interview that followed was to corroborate impairment, if any, in the subject's memory, language, decision making, personal hygiene, financial handling, and social/communal activities. Where positive response was obtained, the mode of onset, pattern of progression, presence of physical incapacitation, and treatment where applicable were documented in a structured manner. Presence of major depressive disorder was screened for using the 15‐item Geriatric Depression Scale (van Marwijk et al. 1995).

Cognitive assessment was carried out using the previously validated IDEA cognitive screen (Gray et al. 2014; Paddick et al. 2015). It tested 10‐word learning (repeated three times), orientation, verbal fluency, abstract reasoning, delayed recall and praxis. The psychometric properties in Nigeria were 100% and 96.3% sensitivity and specificity, respectively, for cut‐off score of ≤7. The interclass correlation (Cronbach's *α*) was 0.741 and a positive predictive value of 75.0% with an Area under Receiver Operation Characteristics curve of 0.99. The details of the validation have been published elsewhere (Paddick et al. 2015). Brief examination for limb paralysis, involuntary movements, gait abnormality, visual and hearing impairment was carried out on all study participants.

### Second‐stage assessment

This took place between January and February 2014. The following criteria were used for selecting individuals for second stage assessment: (1) documentation of impairment on any cognitive domain; (2) informants' positive response to decline in cognitive ability; and (3) Score below 8 on the IDEA screening tool. In addition, 5% of study participants with cognitive scores above the cut‐off value of 7 and who did not report of functional decline were selected by simple random technique using computer‐generated table to check for consistency of diagnosis and for quality control.

**Figure 1:**
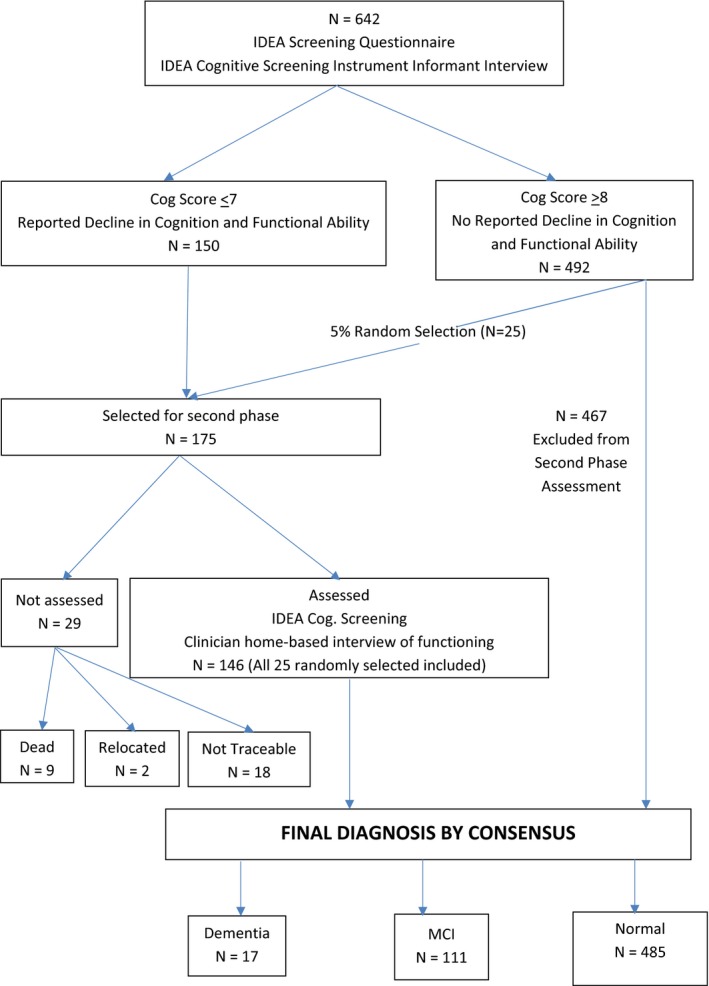
Flow diagram of study participants.

The selected individuals were visited at home by the Research Doctor and/or the Research Nurse and reassessed for cognitive performance. They were unaware of the cognitive scores. Functional competence was assessed using the CHIF (Clinician home‐based assessment of impairment in functioning). CHIF was developed and validated during the Indianapolis–Ibadan Study of Dementia (Hendrie et al. 2006) and later used in the Ibadan Study of Aging (Gureje et al. 2006). Figure 1 shows the flow diagram of the study.

### Diagnosis

All the information obtained was utilized to inform a diagnosis in each case. Two research doctors (AO, BA) independently diagnosed the cases and reached consensus if there was any discordance. The diagnostic categories were Normal, MCI and Dementia according to DSM‐IV, NINCDS‐ADRDA, NINDS‐AIREN, Peterson's criteria (McKhann et al. 1984; Roman et al. 1993; American Psychiatric Association, 1994; Petersen et al. 2001) Sub‐typing of dementia was done as specified in these criteria. Major depressive disorder was suspected to be present when the Geriatric Depression Scale (GDS) score was 10 or higher (van Marwijk et al. 1995).

### Data analysis

Frequency count was done for all variables. We used the SPSS (Statistical Packages for the Social Sciences) version 20 for all analyses. We compared medians (interquartile ranges) using the Kruskal–Wallis test and carried out post hoc testing using the Dunn's method. Categorical variables were compared using the chi‐square test. We calculated age adjustment using the World Health Organization world population estimates (WHO 2015). CI (Confidence intervals) were calculated as appropriate and probability value <0.05 was considered statistically significant. Multivariate Logistic regression model was designed to determine socio‐demographic variables independently associated with neurocognitive impairment with odds ratio, *P*‐values and 95% CI reported.

## Results

Six hundred and forty‐two individuals aged 65 years or over completed the first‐stage assessment out of whom, 175 participants were selected for second stage assessment. However, 29 of them could not be assessed because nine had died, two relocated and 18 could not be traced. The participants that were not assessed were significantly older (Mean age = 83.6 ± 12.1 years vs. 72.9 ± 8.5 years) and had lower frequency of those who were educated (10.3% vs. 22.6%). However, the diagnostic categories based on first‐stage assessment data were not significantly different from those that were assessed (*χ* ^2^ = 3.74; *P* = 0.15).

The 613 participants that completed the study included 427 (69.7%) female and 186 (30.3%) male. The mean age of the cohort was 72.9 ± 8.5 years and by gender, the mean ages were 72.5 ± 8.3 and 73.7 SD 8.9 years for female and male, respectively. There was no statistically significant difference in mean ages by gender (*F* = 1.7; *P* = 0.10). Four hundred and 27 (69.7%) were in the 65–74 years age group, 117 (19.1%) were in the 75–84 year group while the remaining 69 (11.2%) were aged 85 years and above. Overall, three participants (0.5%) had scores that were suggestive of Major Depressive Disorder and two of them had normal cognition while one met the criteria for dementia diagnosis.

Four hundred and eighty‐five participants (79.1%) had normal cognition and these included the 25 subjects that were selected for second‐stage assessment, while 128 (20.9%) had neurocognitive impairment. The latter comprised 111 (18.1%) with MCI and 17 (2.8%) with dementia. Table 2 shows a comparison of the demographic variables and test scores between the three diagnostic categories. Gender distribution showed a significantly higher frequency of female subjects in the MCI group while the dementia group had a male preponderance (*χ* ^2^ = 11.6; *P* = 0.003). The participants diagnosed with normal cognition were the youngest and their median age of 70 years was significantly lower than those diagnosed with neurocognitive impairment. Those diagnosed with dementia were the oldest, however, their median age was not significantly different from those with MCI (*P* = 0.1) in post hoc analysis. The MCI participants had the least years of education which was significantly lower than for those with dementia and normal cognition (*P* < 0.0001) on post hoc analysis. There was no significant difference in the median years of education between those with normal cognition and those diagnosed as demented. There was a gradient of total cognitive scores from normal through MCI to dementia and the differences between the groups were statistically significant (*P* < 0.0001) (Table 1).

**Table 1:** Comparison of socio‐demographic and test variables of study participants according to diagnostic categories^a^

Variable	Normal *N* = 485	Mild cognitive impairment *N* = 111	People with dementia *N* = 17	Statistics
Median age in years (IQR)	70 (67–73)	78 (69–85)	82 (80–87)	H(2) = 58.8, *P* < 0.0001
% Female	68.3	80.2	41.2	X^2^ = 12.8; *P* = 0.002
Median years of education (IQR)^b^	0 (0–6)	0 (0–0)	0 (0–1)	H(2) = 12.1; *P* < 0.0001
% Educated^a^	33.6	13.5	29.4	X^2^ = 17.4; *P* < 0.001
Median total cog scores (IQR)	15 (12–16)	11 (8–14)	8 (4–12)	H(2) = 61.9; *P* < 0.0001

a Interquartile Range in parentheses.

b No difference between participants with dementia and normal participants.

Any Education.

The standardized, age‐adjusted prevalence estimates for dementia and MCI were 2.9% (95% CI = 1.6–4.4%) and 18.4% (95% CI = 14.9–21.9%), respectively. The standardized estimates of neurocognitive impairment according to age‐groups are shown in Table 2. The sub‐types of dementia documented were: probable AD (Alzheimer's diseases) 10 cases (58.8%), mixed dementia three cases (17.6%), and two cases each of VD (vascular dementia) and Parkinson's disease with dementia. The mixed dementia group included one participant who had suspected Major Depressive Disorder along with features consistent with possible AD. For MCI, the sub‐types were: 47 cases of single‐domain amnestic type (42.3%); 45 cases of multi‐domain amnestic type (40.5%), 18 cases of single‐domain nonamnestic type (16.2%) and one case of multi‐domain nonamnestic type (0.9%).

**Table 2:** Cognitive classification according to age‐group

Age‐group (in years)	Normal	CI	Dementia	Total
65–74	374	50	3	427 (69.7)
		**11.7%**	**0.7%**	
		**CI = 8.5–14.9%**	**CI = 0.1–1.5%**	
75–84	80	30	7	117 (19.1)
		**25.6%**	**6.0%**	
		**CI = 16.5–34.8%**	**CI = 1.5–10.4%**	
>85	31	31	7	69 (11.2)
		**44.9%**	**10.1%**	
		**CI = 29.1–60.7%**	**CI = 2.6–17.7%**	
Total	485 (79.1)	111 (18.1)	17 (2.8)	613 (100.0)
		**18.4%**	**2.9**	
		**CI = 14.9–21.8%**	**CI = 1.6–4.4%**	

Standardized age‐adjusted prevalence estimates in bold with confidence limits,

Female participants and the uneducated participants were not at increased odds of cognitive impairment (*P* = 0.236 and *P* = 0.159, respectively) on regression analysis. Participants between the age of 75 and 84 years and those over 85 years were three times and six times, respectively, more likely to develop cognitive impairment when compared to those between 65 and 74 years. These are shown in Table 3.

**Table 3:** Logistic regression of factors associated with neurocognitive impairment

	OR	95% CI	*P*‐value
Gender			
Male	–	–	–
Female	1.4	0.8–2.4	0.236
Age group			
65–74	–	–	–
75–84	3.2	1.9–5.2	0.001^a^
Over 85	6.2	3.2–11.9	
Education			
Educated	–	–	–
No Education	1.5	0.8–2.7	0.159

OR, Odds Ratio.

a Statistically significant.

## Discussion

The results of this study showed that approximately one in five older persons in this rural community had major neurocognitive impairment. The age‐adjusted prevalence estimate of dementia of 2.9% is in the lower range of rates published in the literature for SSA but was slightly higher than the prevalence figures for Idikan community in Ibadan metropolis (Hendrie et al. 1995), Zaria in Northern Nigeria (Yusuf et al. 2011) and Djija in Benin Republic (Guerchet et al. 2010). Lalupon is very close to Ibadan city hence, it is not surprising that the prevalence estimates in the two communities are similar. We utilized a two‐phase study design and our research methodology was similar to that of the Indianapolis‐Idikan study except that we used the IDEA study screening instrument rather than the Community Screening Instrument for Dementia. A much higher prevalence estimate than we obtained was reported by Gureje et al. (2006) in a wider geographical area in South‐western Nigeria and so far, is the highest reported rate in Africa. That rate from that Ibadan Study of Aging could have been overestimated because the main cognitive assessment was based on performance on the 10‐word list score.

Wide variation in dementia prevalence has been reported globally due to differences in study methodology and the age‐group investigated. High rates of dementia have been reported from two countries in Central Africa (Guerchet et al. 2010; Paraïso et al. 2011; Mbelesso et al. 2012) while in Hai District, Tanzania, the prevalence was 6.4% in individuals aged 70 years and above (Longdon et al. 2013). Comparing our results with prevalence estimates from other regions of the world, the lowest rate thus far, was 0.3%. That was reported from a study in rural India while figures obtained from studies in Latin American countries ranged between 6.2% in Venezuela to 12.6% in Cuba (Lilbre Rodriguez et al. 2008). Those studies utilized the 10/66 diagnostic algorithm which appeared to give higher rates than the DSM‐IV criteria as illustrated by data from Hai in which a dementia prevalence estimate of 21.6% was obtained, using the 10/66 criteria compared with 6.4% based on DSM IV criteria (Paddick et al. 2013). However, differences in study methodology (case classification and diagnosis) make direct comparison of results very difficult.

Our results showed a progressive increase in prevalence with age as had been shown in all community‐based studies of dementia (Ferri et al. 2005; World Health Organization, 2012; Lekoubou et al. 2014; Olayinka and Mbuyi 2014). For those above 85 years of age, the upper limit of the adjusted prevalence was 17.7% which implied that with a much older cohort, even in Lalupon, the overall prevalence could have been higher than 2.9%. The individuals that were not assessed were older and probably could conceivably have increased the number of cases diagnosed with dementia. However, only two of these individuals reported some functional impairment which was corroborated by carers during the first‐stage assessment. Our analysis showed that the distribution of the diagnostic categories was not significantly different from the prevalence estimates even with their addition. We observed a male preponderance among those diagnosed with dementia which is different from the gender distribution in Idikan, Ibadan where a significantly higher proportion of the demented subjects were female (Hendrie et al. 1995; Ogunniyi et al. 2000). Gender association of dementia has not been a consistent finding in all studies (Ferri et al. 2005; World Health Organization, 2012).

Probable AD was the predominant type of dementia (58.8%) in this study and this agreed with the finding from other studies (Ferri et al. 2005; World Health Organization, 2012; Mavrodaris et al. 2013; Olayinka and Mbuyi 2014). If individuals diagnosed with mixed dementia were included, the frequency of AD would have risen to 76.4% which is still within the range usually reported in community‐based studies. Our results showed that dementia associated with Major Depressive Disorder was relatively uncommon and VD accounted for about 11.8% in the cohort.

With regard to MCI, our age‐adjusted prevalence estimate of 18.4% lies within the range of 6.5% to 25% reported from studies in SSA (Mavrodaris et al. 2013; Lekoubou et al. 2014; Olayinka and Mbuyi 2014). The estimate was 44.9% with the upper confidence limit reaching 60.7% for persons older than 85 years which implied that over half of very old individuals may have MCI. Our study showed that age was the only significant risk factor for major neurocognitive impairment in the rural community studied and neither gender nor years of education was associated with increased risk.

The strength of our study is the total community study of individuals aged 65 years and above utilizing a door‐to‐door approach. This was the design used in all studies that determined prevalence estimates of dementia in Nigeria. This ensured that we did not miss any cases that might been concealed at home The lack of refusals was ascribed to the close ties with the community by the Department of Community Medicine and we engaged community health workers resident in the community for the first‐stage assessment. Failure to do neuroimaging was, however, a limitation for accurate diagnosis of the sub‐types of neurocognitive impairment and identification of possible treatable conditions.

## Conclusion

Our study showed that about one out of every five older persons in Lalupon community had major neurocognitive disorder with MCI predominating. The age‐adjusted prevalence estimates of dementia and MCI were within the figures available in literature. The study participants with dementia were older while those with MCI had lower educational attainment. More community‐based studies are needed for rate comparison and for teasing out putative risk factors for neurocognitive impairment in SSA.

## Conflict of Interest

None declared.
